# Monocular and Binocular Temporal Visual Perception of Infantile Nystagmus

**DOI:** 10.1038/s41598-020-61914-2

**Published:** 2020-03-18

**Authors:** Avital Moshkovitz, Maria Lev, Uri Polat

**Affiliations:** School of Optometry and Vision Sciences, Faculty of Life Sciences, Bar-Ilan University, Ramat Gan, Israel

**Keywords:** Sensory processing, Striate cortex

## Abstract

Contrast sensitivity is mostly used as a tool for testing aspects of visual functions. Infantile nystagmus is a pathological phenomenon that affects the spatial-temporal visual functions due to spontaneous oscillating movements of the eyes. We examined the spatial-temporal aspects of nystagmus perception, aiming to investigate the mechanisms underlying the deterioration of their visual performance. We tested the monocular and binocular contrast sensitivity of nystagmus and normally sighted subjects by measuring contrast detection of a Gabor target with spatial frequencies slightly above the cutoff threshold of each subject (nystagmus ~3; controls = 9cpd; presentation times 60–480 ms). The dominant eye of nystagmus revealed large differences over the non-dominant eye, highlighting the superiority of the dominant over the non-dominant eye in nystagmus. In addition, binocular summation mechanism was impaired in majority of the nystagmus subjects. Furthermore, these differences are not attributed to differences in visual acuity. Moreover, the visual performance in nystagmus continue to improve for longer presentation time compared with controls and was longer in the poor eye. Since the results are not due to differences in eye movements and strabismus, we suggest that the differences are due to developmental impairment in the visual system during the critical period.

## Introduction

Contrast sensitivity refers to the ability to detect fine changes in luminance. It is considered to be one of the most standard measures for evaluating spatial processing and visual functions; it develops from birth to a fully mature status at the age of 8 to 19 years old^1^. The quality of contrast sensitivity is affected by both retinal and cortical factors; this depends on the development of the visual system^2^ and the optical quality of the eye. It was also shown that contrast sensitivity decreases with age^3–5^. Additional factors affecting the contrast sensitivity function (CSF) include the spatial parameters of the target such as size and color^6,7^, orientation^8,9^, illumination^10,11^, presentation time^12^ and eye movements^11,13^.

Visual acuity is a measure of the spatial resolution associated with visual performance. Normal visual acuity is defined as the ability to discriminate between two contours separated by 1 arc minute (1.75 mm) from 6 meters. Although visual acuity is frequently used as the main factor of visual functions, sometimes it does not fully capture the functional and visual abilities. Sometimes a deficit in contrast sensitivity was found, whereas visual acuity was reported as normal. The difference between visual acuity and contrast sensitivity is especially apparent in clinical cases such as myopia, early cataracts^14^, congenital glaucoma^14^, and the initial stages of diabetic retinopathy^15^.

In normal visual development the brain combines the information it receives from both eyes, resulting in superior visual binocular performance over monocular performance, termed binocular summation. In contrast sensitivity, binocular summation refers to the equation of CSbin = sqrt (CSright^2^ + CSleft^2^). At high spatial frequencies, the summation is higher^16^. In addition, the longer the presentation time, the higher the summation will be. Studies on normally sighted populations with normal development agree that binocular summation occurs when no difference exists between the eyes’ performance. Thus, the most accepted theory of binocular summation suggests that binocular summation is absent or minor in cases of abnormal binocular development such as amblyopia, and that it decreases as the magnitude of the difference between the eyes increases^16–21^. However, some studies found evidence of binocular summation in cases of amblyopia^22,23^.

In addition to spatial parameters, such as contrast sensitivity and visual acuity, visual function can also be evaluated regarding its temporal processing abilities. Generally, visual functions improve as the stimulus exposure time increases – up to a certain point. The location of the transition response over time, known as the critical duration, is defined as the point at which further stimulus presentation time does not contribute to enhanced performance^6,24^. In some studies, critical duration refers to the time in which contrast sensitivity reaches 90% of the maximal value^24,25^. Critical duration can be influenced by spatial and temporal parameters. Studies suggest that the period of temporal integration is affected by spatial frequency; the higher the spatial frequency is, the longer will be the time needed for integration^19^. In the literature, the range of critical duration is between 160 and 200^19,26^. In addition, critical duration can change as a result of cortical deprivation in cases such as amblyopia^27–31^ or neurological disease^29^.

Nystagmus is a form of spontaneous oscillation of the eyes, which results in excessive motion of images on the retina, accompanied by poor vision including a reduction in visual acuity^32–35^. In normal fixation, the eyes are not still; they move at a velocity of 3 minutes of arc in one second^36^. Infantile nystagmus (IN) is accompanied by reduced vision in both eyes during the critical periods of life^37–39^. The prevalence of infantile nystagmus is 1 to 1000–6000^34,35^. The most common form of infantile nystagmus is afferent nystagmus; it is caused by impairment of central vision in early life, e.g., albinism or congenital cataract^35,40^. Albinism is a heterogeneous group of congenital disorders affecting melanin synthesis. A few weeks after birth, nystagmus can appear in albino subjects^41^. In research investigating nystagmus, albinos are one of the main participants^42–44^. In addition, if efferent, it is an idiopathic nystagmus, without any involvement of ocular or systemic pathology. The onset is about 2–3 months after birth and it persists throughout life^45,46^. In cases of nystagmus, contrast sensitivity is reduced^35,40,47–49^.

Vision impairment of cortical origin, such as amblyopia, is characterized by deficits in normal processing such as binocular summation and critical duration. In this study we investigated how binocular and monocular processing was affected in IN due to visual impairment. We hypothesized that in nystagmus, similarly to amblyopia, we would observe absent or diminished binocular summation from the two eyes, and that the critical duration differs from normal sighted vision.

## Results

Ten nystagmus and ten normally sighted subjects underwent contrast sensitivity tests. This experiment investigated temporal contrast sensitivity processing, measured in five blocks in which the stimulus duration was set to 60, 120, 240, 320, and 480 ms. The measurements were acquired under both monocular and binocular conditions. Eight nystagmus subjects performed 4 repetitions of the experiment (subjects NYS 1–8). One subject repeated the experiment three times (subject NYS-9). Another subject repeated the experiment two times (subject NYS-10). All normally sighted subjects repeated the test twice (Table 1).

**Table 1:** Clinical optometric information of nystagmus subjects: R right eye, L left eye, XP exophoria, XT exotropia, ET esotropia, IN intermittent, AL alternating, and OCA Oculocutaneous albinism.

Subject		NYS-I	NYS-II	NYS-III	NYS-IV	NYS-V	NYS-VI	NYS-VII	NYS-VIII	NYS-IX	NYS-X
Type		Motor nystagmus	OCA	OCA	OCA	OCA	OCA	OCA	OCA	Motor nystagmus	OCA
Sex		F	M	F	M	F	M	F	M	M	M
Age		25	3	43	22	31	31	31	19	28	37
Eyes misalignment [∆ D]		6 XP	10 LXT	8 XP	6 INALET	8 INXT	14 ALET	10 ALXT 3R HYPER	4 LET 4 L.HYPOT	7 XP	10 RET
VA: Far [logMar]	L	0.16	1.02	0.8	0.34	0.66	1.06	1.3	0.82	0.64	0.82
	R	0.04	0.86	0.72	0.14	0.74	0.92	1.36	0.72	0.64	0.88
	BI	0.08	0.96	0.66	0.12	0.6	0.86	1.3	0.74	0.62	0.82
VA: Near [logMar]	L	0.18	0.92	0.88	0.38	0.66	1.18	1.22	0.86	0.72	0.7
	R	0.1	0.82	*0.9	0.2	0.7	0.82	1.22	0.84	0.64	0.84
	BI	0.02	0.82	0.92	0.04	0.68	0.9	1.12	0.86	0.64	0.66
Dominant eye		R	R	R	R	L	R	L	R	R	L
Correction	L	+2.50–1.75×180	+4.25−5.25 × 165	+5.00−2.50 × 180	+1.00−0.50 × 170	+2.50−4.75 × 175	+4.50−0.50 × 30	−1.75−1.25 × 175	−0.50−1.00 × 10	−0.50−0.75 × 175	+3.50−2.50 × 180
	R	+1.00	+3.75−4.50 × 15	+4.50−3.00 × 180	pl-0.50 × 150	+2.75−5.50×10	+4.50−2.00 × 170	−3.00−0.75 × 160	Pl −2.25 × 10	−1.00−1.75 × 35	+1.25−1.75 × 175
Previous correction		Yes	Yes	No	Yes	Yes	Yes	No	Yes	No	No
Worth 4 Dots		Fusion	Fusion	Fusion	Fusion	R Suppression	Dipplopia	Dipplopia	AL suppression	L Suppression	R Suppression
Spatial frequency [cpd]		8	2	1	4	3	1	0.5	2	4	2

*Visual acuity was also measured at the distance of the experiment L: 0.82 R: 0.78 BI: 0.78 (logMar).

To evaluate repetition reliability, and more importantly, to rule out inconsistent measurements (outliers), we calculated the coefficient of variation of the results of each subject per time condition. The tests were highly reliable, as evident by the small standard deviation value for the R eye (0.31), L eye (0.32), and for binocular (0.32). Moreover, no significant differences, in terms of reliability, were found between the eyes, as confirmed by ANOVA (p = 0.42).

**Figure 1:**
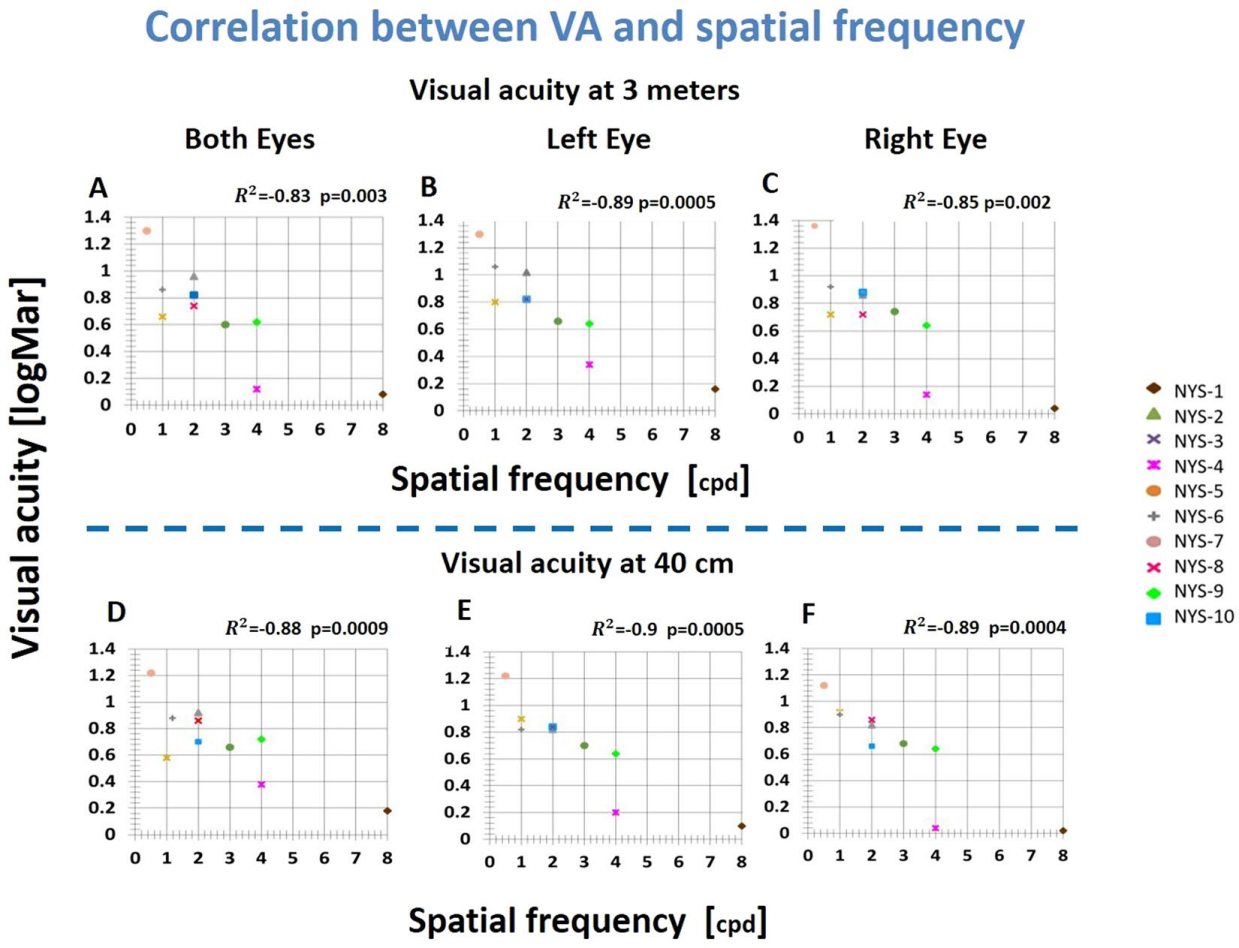
Scatter plots of visual acuity (at 40 cm and 300 cm) and the measured spatial frequency. The goodness of the correlation is reported in the correlation coefficient and statistical results (p value). In R². A higher correlation was obtained at near visual acuity (R² = 0.88–0.9, p < 0.001), in agreement with the distance of the exam.; the psychophysical exam was performed under the same conditions (near).

The spatial frequencies of the Gabor patches were adjusted such that for each subject, the chosen spatial frequency was slightly above the cutoff frequency. Among the IN subjects, the spatial frequency varied from 0.5 to 8 cpd (see Table 1 (2.75 ± 2.2, mean ± SD)), whereas for normally sighted subjects it was fixed at 9 cpd. For IN, the correlations between the spatial frequencies and visual acuity for monocular and binocular conditions for near and far distances are presented in Fig. 1. The correlations at near distances between right, left, and both eyes and spatial frequencies were similar and high (r² = 0.89, 0.9, 0.88, respectively p < 0.001). For the control subjects, since the spatial frequency was constant (9 cpd) and minimal variability existed in visual acuity, no correlation was performed.

Pearson’s correlation analysis was provided by MATLAB. The Pearson analysis highlighted the high correlation between the visual acuity and the spatial frequency.

The contrast sensitivity averages were calculated for IN and normally sighted subjects for each temporal condition. According to the literature, infantile nystagmus subjects have variability in their visual performance^38,47^. Hence, we normalized the results of each temporal condition by the results of the longest stimulus duration (480 ms) under binocular conditions, for both normal and IN subjects. The contrast sensitivity data were then fitted to an exponential curve (described in the Methods section) to further analyze the trend and extract critical duration (plateau and time constants).

### Nystagmus subjects

**Figure 2:**
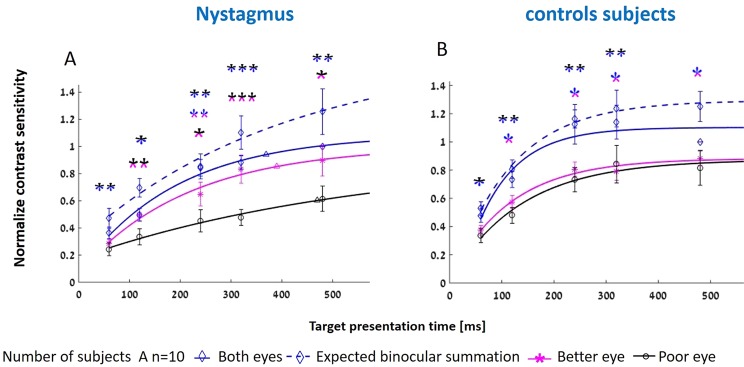
Normalized contrast sensitivity for Infantile nystagmus (IN) and control subjects for varying presentation times. The fitting curve is described in the Methods section. (**A**) Results for the better eye were determined by the dominant eye for IN subjects. (**B**) The same as in A. for control subjects. Each line is a fit to the monocular or binocular conditions of measured contrast sensitivity. Binocular, better, and poor eye are denoted in blue, magenta, and black solid lines, respectively. Dashed blue lines denote the square root calculation of binocular summation (CSbin = sqrt (CSpoor^2^ + CSbetter^2^)). Error bars refer to the standard error of the mean. Critical duration is denoted by triangular symbols in corresponding colors. Statistical significance was indicated: *p ≤ 0.05 **p ≤ 0.01 ***p ≤ 0.001. The two halves of the asterisk are indicated in colors corresponding to the conditions that were statistically compared (both/better/poor eyes).

Normalized contrast sensitivity results of IN are presented in Fig. 2. Individual normalized contrast sensitivity results of nystagmus subjects along with critical durations calculation presented in supplementary section (Fig. S1). As expected, contrast sensitivity increases with increasing stimulus duration, showing a minimal value at 60 ms and reaching a maximal value at 480 ms for all eye conditions ((max ± SE) L: 0.71 ± 0.09. R: 0.8 ± 0.11. B: 1.00 ± 0.00)). The increase in contrast sensitivity in the L eye is slower than in the R eye and that the binocular viewing condition did not reach saturation even for the longest investigated duration. the R/L ratio was 1.33 ± 0.27 (mean ± SE). However, no statistical differences were found (Table 2) between the R and L eyes (2-way ANOVA, p = 0.23) with eyes (R, L) and the time presentation [ms] (60, 120, 240, 320, and 480); thus, we assumed that the large error bar of the R eye compared to the L eye implies on strong differences between eyes. Some previous studies investigated infantile nystagmus and categorized the results as better and poor eyes^50,51^. Thus, we further attempted to distinguish between the contrast sensitivity of each eye with optometric measures such as VA and dominant eye (Table 1), elaborated in the following section.

**Table 2:** Statistical information on normalized contrast sensitivity results in IN subjects.

Statistical chart of IN subjects (normalized results)						
Time Eye	60 ms	120 ms	240 ms	320 ms	480 ms	ANOVA
*Right -Left*	0.24 ± 0.03, 0.29 ± 0.05 P = 0.37	0.42 ± 0.05, 0.41 ± 0.06 P = 0.89	0.58 ± 0.09, 0.52 ± 0.08 P = 0.51	0.74 ± 0.1, 0.57 ± 0.06 P = 0.2	0.8 ± 0.11, 0.71 ± 0.09 P = 0.52	P = 0.234
*Binocular Viewing-Right*	0.36 ± 0.05, 0.24 ± 0.03 P = 0.01	0.5 ± 0.05, 0.42 ± 0.05 P = 0.21	0.84 ± 0.06, 0.58 ± 0.09 P = 0.004	0.88 ± 0.05, 0.74 ± 0.1 P = 0.09	1.00 ± 0.00, 0.8 ± 0.11 P = 0.12	P = 2.8289e-04
*Binocular Viewing -Left*	0.36 ± 0.05, 0.29 ± 0.05 P = 0.05	0.5 ± 0.05, 0.41 ± 0.06 P = 0.18	0.84 ± 0.06, 0.52 ± 0.08 P = 0.01	0.88 ± 0.05, 0.57 ± 0.06 P = 0.003	1.00 ± 0.00, 0.71 ± 0.09 P = 0.01	P = 8.2324e-08
*Poor-Better*	0.24 ± 0.05, 0.288 ± 0.02 P = 0.355	0.33 ± 0.06, 0.9 ± 0.08 P = 0.008	0.45 ± 0.09, 0.65 ± 0.06 P = 0.012	0.48 ± 0.07, 0.83 ± 0.06 P = 0.0007	0.62 ± 0.1, 0.896 ± 0.08 P = 0.028	p = 5.6385e-06
*Binocular Viewing-Better*	0.36 ± 0.04, 0.29 ± 0.02 P = 0.052	0.5 ± 0.05 0.495 ± 0.03 P = 0.967	0.84 ± 0.06, 0.65 ± 0.06 P = 0.01	0.88 ± 0.05, 0.83 ± 0.06 P = 0.41	1.00 ± 0.0, 0.896 ± 0.08 P = 0.243	P = 0.01
*Binocular Viewing-Poor*	0.36 ± 0.04, 0.24 ± 0.05 P = 0.008	0.497 ± 0.05, 0.33 ± 0.06 P = 0.02	0.84 ± 0.06, 0.45 ± 0.09 P = 0.004	0.88 ± 0.05, 0.48 ± 0.07 P = 6.22E-05	1.00 ± 0.00, 0.615 ± 0.1 P = 0.005	P = 9.5911e-11.
*Binocular Summation-Binocular Viewing*	0.47 ± 0.07, 0.36 ± 0.04 P = 0.13	0.7 ± 0.07, 0.5 ± 0.05 P = 0.04	0.85 ± 0.09, 0.84 ± 0.06 P = 0.9	1.1 ± 0.12, 0.88 ± 0.05 P = 0.07	1.3 ± 0.17, 1.00 ± 0.00 P = 0.16	P = 0.004

Each column represents a different presentation time and each row represents the two conditions with which the statistical analysis is compared. The evaluation was done using two-tailed paired t-test and 2-way ANOVA. Binocular summation refers to the squirt root calculation of the better and poor eyes.

### Comparison of contrast sensitivity between eyes

Optometric measures for determining the better eye (dominant eye and better visual acuity) are well established in the field of psychophysics and were utilized in this study. The resulting better and poorer eye’s visual acuity is presented in Table 3. The mean differences between the poor-better visual acuity were 0.1 ± 0.02 logMar (mean ± SE) for both near and far distances. Furthermore, the differences in visual acuity under the new distinction of poor-better were statistically significant for the two distances (far, p = 0.0003; near, p = 0.02. paired two-tailed t-test; Table 3). Differences between visual acuity without this distinction (R-L eyes) of IN visual acuity were not statistically significant at all distances [Far: (R: 0.76 ± 0.1, L:0.7 ± 0.12, p = 0.096); Near: (R: 0.77 ± 0.1, L0.71 ± 0.1, p = 0.185, paired two-tailed t-test) (mean ± SD, p value)] (Table 3). The resulting better eye, based on the better visual acuity, was compatible with the subjects’ dominant eye.

**Table 3:** Clinical optometric information of normal subjects.

Subject		SUB-I	SUB-II	SUB-III	SUB-IV	SUB-V	SUB-VI	SUB-VII	SUB-VIII	SUB-IX	SUB-X
Sex		F	F	M	F	F	F	M	F	F	F
Age		27	32	32	26	36	23	26	25	26	27
Eyes misalignment [∆ D]		6 XP	5 XP	Ortho	Ortho	Ortho	Ortho	5 EP	8 XP	3 XP	4 EP
VA: Far [logMar]	L	−0.18	−0.18	−0.08	−0.26	−0.04	−0.12	−0.02	−0.2	−0.08	−0.1
	R	−0.16	−0.12	−0.08	−0.26	0.02	−0.08	0.00	−0.1	−0.1	−0.08
	BI	−0.24	−0.14	−0.06	−0.28	−0.04	−0.2	−0.08	−0.18	−0.1	−0.16
VA: Near [logMar]	L	−0.1	−0.12	0.00	−0.12	−0.1	−0.08	−0.1	−0.08	−0.04	−0.1
	R	−0.1	−0.12	0.00	−0.2	−0.1	−0.08	−0.02	−0.02	0.02	−0.1
	BI	−0.1	−0.12	−0.08	−0.14	−0.1	−0.16	−0.1	−0.08	−0.04	−0.1
Dominant eye		R	R	R	L	R	R	L	L	L	R
Correction	L	pl	−4.50−1.00 × 175	−4.50−1.00 × 170	−0.25	−0.50−0.50 × 90	−2.50−0.25 × 166	pl	−0.25	−2.25−0.25 × 97	pl
	R	pl	−5.00−0.75 × 005	−4.25−1.00 × 180	−1.50	0.50−0.50 × 90	−3.00−0.25 × 143	pl	−0.25	−3.25	pl
Worth 4 Dots		Fusion	Fusion	Fusion	Fusion	Fusion	Fusion	Fusion	Fusion	Fusion	Fusion
Spatial frequency [cpd]		9	9	9	9	9	9	9	9	9	9

^XP^Exophoria ^EP^Esophoria.

Thus, we assigned the results of the contrast sensitivity to poorer and better eyes, based on the difference in visual acuity. The results are presented in Fig. 2. Each line represents a calculated curve as described in the Methods section; they were fit to the monocular or binocular conditions of the measured contrast sensitivity. Binocular, better, and poor eyes are denoted by blue, magenta, and black solid lines, respectively. We again see an increase in contrast sensitivity as a function of increasing the presentation time of the target. The results of the better eye are superior to the poor eye. To quantify this superiority, we divided the better eye by the poor eye for all temporal durations. The results are 1.18, 1.99, 1.89, 2.1, and 1.8 for 60, 120, 240, 320, and 480 ms, respectively. Differences between eyes were statistically significant p < 0.0001, confirmed by 2-way ANOVA with eyes (Better/Poor) and presentation time [ms] (60, 120, 240, 320, and 480). (See Table 2 for more statistical information about temporal data). Thus, the results clearly show a dichotomy between the eyes, where one eye is superior to the other. Note that this effect is not apparent by a standard right-left eye distinction of the visual acuity between the eyes. Finally, the critical duration was calculated using the new distinction, yielding durations of 470, 389, and 369 ms for poor, better, and binocular, respectively. However, the individual results showed variability in critical duration as dependency of the performance of the poor or better eyes.

### Binocular summation

Models of binocular summation suggest that in cases of large differences between the eyes, binocular vision will be closer to the better eye. We investigated this issue and calculated the binocular ratio results compared to the better eye; the results show that for the binocular/better eye the average ratio for all durations was 1.2 ± 0.1 (60,1.3 ± 0.1; 120,1 ± 0.1; 240,1.4 ± 0.1; 320,1.1 ± 0.08; 480,1.2 ± 0.1; mean ± SE). These ratios show some priority of binocular viewing over the better eye; the effect was confirmed to be statistically significant (p = 0.01), by 2-way ANOVA with eyes (Binocular/Better) and presentation time [ms] (60, 120, 240, 320, and 480). For more temporal statistical information, see Table 2.

When examining the superiority of binocular viewing over the poor eye condition, we found a larger average ratio of 2.17 ± 0.37 (60,2.0 ± 0.4; 120,1.88 ± 0.23; 240,2.7 ± 0.5; 320,2.14 ± 0.24; 480,2.1 ± 0.37 mean ± SE). This robust superiority of binocular viewing was also found to be highly significant (p < 0.001) by 2-way ANOVA with eyes (Binocular/Poor) and presentation time [ms] (60,120,240,320,480) (Table 2).

Thus, according to our results, this clinical approach of binocular viewing shows much more similarity for the better eye compared with the poor eye. These results are consistent with binocular models suggesting that binocular viewing is determined mainly by the good eye when large differences exist between eyes.

### Binocular summation by a neural mechanism

The calculation presented above for binocular/better eyes is adequate for determining clinical binocular superiority over the monocular results. The clinical binocular superiority was found here resemble more the better eye contrast sensitivity results, however, with significant differences and with better performance. For better understanding the neural mechanism of binocular summation and the proper neural convergence of the summation of the two eyes in infantile nystagmus, we examined the summation calculation based on the square root calculation, denoted as a dashed blue line in Fig. 2A,B.

When comparing the results of the calculated predicted binocular summation to the binocular performance of the nystagmus subjects, the results indicated that for the calculated ratio of binocular summation (prediction/measured), the average ratio for all durations was 1.27 ± 0.15 (60,1.32 ± 0.17; 120,1.5 ± 0.2; 240,1 ± 0.1; 320,1.25 ± 0.1; 480,1.26 ± 0.17; mean ± SE). These ratios show that the expected binocular summation is higher than the actual binocular viewing, and may imply that the neural binocular summation is intact but is significantly different than the measured binocular summation (p = 0.004) by 2-way ANOVA with eyes; (prediction/measured) and presentation time [ms] (60, 120, 240, 320, and 480). For more statistical information, see Table 2. However, when examining the individual data, four subjects (NYS-1, NYS-3, NYS-5 and NYS-7) show that the measured is at least as good as the predicted, suggesting normal neural and binocular summation, whereas the superiority effect of the strong eye over the poor eye remained intact (see supplementary).

### Normally sighted subjects (controls)

The same analyses as previously described were performed for the ten normally sighted subjects. Information about their clinical optometric exams is presented in Table 4.

**Table 4:** Statistical information of normalized contrast sensitivity results in control subjects.

Statistical chart of normal sighted subjects (normalized results)						
*Time Eye*	*60 ms*	*120 ms*	*240 ms*	*320 ms*	*480 ms*	ANOVA
*Right-Left*	0.34 ± 0.04, 0.37 ± 0.05 P = 0.48	0.54 ± 0.06, 0.51 ± 0.06 P = 0.7	0.72 ± 0.08, 0.81 ± 0.06 P = 0.33	0.85 ± 0.12, 0.78 ± 0.07 P = 0.52	0.88 ± 0.11, 0.82 ± 0.07 P = 0.63	P = 0.91
*Binocular Viewing-Right*	0.48 ± 0.05, 0.34 ± 0.04 P = 0.0004	0.73 ± 0.06, 0.54 ± 0.06 P = 0.001	1.12 ± 0.14, 0.72 ± 0.08 P = 0.003	1.14 ± 0.12, 0.85 ± 0.12 P = 0.003	1.00 ± 0.00, 0.88 ± 0.11 P = 0.31	P = 0.0001
*Binocular Viewing- Left*	0.48 ± 0.05, 0.37 ± 0.05 p = 0.137	0.73 ± 0.06, 0.51 ± 0.06 P = 0.015	1.12 ± 0.14, 0.81 ± 0.06 P = 0.055	1.14 ± 0.12, 0.78 ± 0.07 p = 0.016	1.00 ± 0.00, 0.82 ± 0.07 P = 0.03	P = 4.1564e-06
*Poor-Better*	0.34 ± 0.05, 0.37 ± 0.03 P = 0.31	0.48 ± 0.06, 0.57 ± 0.05 P = 0.02	0.73 ± 0.09, 0.8 ± 0.05 p = 0.38	0.84 ± 0.13, 0.79 ± 0.06 p = 0.92	0.82 ± 0.12, 0.88 ± 0.05 P = 0.18	P = 0.356
*Binocular Summation-Better*	0.48 ± 0.05, 0.37 ± 0.03 P = 0.07	0.73 ± 0.06, 0.57 ± 0.05 P = 0.03	1.12 ± 0.14, 0.8 ± 0.05 P = 0.04	1.14 ± 0.12, 0.79 ± 0.06 p = 0.018	1.00 ± 0.00, 0.88 ± 0.05 P = 0.05	P = 1.3854e-05
*Binocular Summation-Poor*	0.48 ± 0.05, 0.34 ± 0.05 P = 0.014	0.73 ± 0.06, 0.48 ± 0.06 P = 0.001	1.12 ± 0.14, 0.73 ± 0.09 P = 0.006	1.14 ± 0.12, 0.84 ± 0.13 P = 0.003	1.00 ± 0.00, 0.82 ± 0.12 P = 0.17	P = 3.6652e-05
*Binocular Summation-Binocular Viewing*	0.53 ± 0.05, 0.48 ± 0.05 P = 0.33	0.8 ± 0.07, 0.73 ± 0.06 P = 0.2	1.16 ± 0.06, 1.12 ± 0.14 P = 0.73	1.24 ± 0.13, 1.4 ± 0.12 P = 0.54	1.25 ± 0.1, 1.00 ± 0.00 P = 0.04	P = 0.07

Each column represents different presentation times and each row represents the two conditions with which the statistical analysis is compared. The evaluation was done using two-tailed paired t-test and 2-way ANOVA. Binocular summation refers to the squirt root calculation of the better and poor eyes.

### Effect of presentation time

Results of binocular, right, and left eyes for five durations were calculated for the normalized results and are denoted as blue, red, and green lines, respectively, in Fig. 2B. As shown for the nystagmus subjects, contrast sensitivity increases as a function of presentation time, reaching a maximal value at 480 ms. Interestingly, for binocular vision the maximum value was reached at 320 ms. The maximal values of the normalized results: L eye: 0.82 ± 0.07; R eye: 0.87 ± 0.11; both eyes: 1.14 ± 0.12. (Max ± SE) critical duration for binocular, right, and left eyes are 196, 310, and 245 ms, respectively.

### Binocular summation

The average R/L eye ratio was 1.05 ± 0.14 (mean ± SE), indicating no differences between the performance of the R and L eyes (p = 0.91), confirmed by 2-way ANOVA with eyes (R, L) and presentation time [ms] (60, 120, 240, 320, and 480 (Table 4). This implies that no differences between the eyes were found. Hence, the binocular summation calculation is valid. Average binocular summation (Binocular/Average of both monocular) was 41% (60,41% ;120,45%; 240,47%; 320, 47%; and 480, 25%). For more statistical information, see Table 4.

To test the neural binocular summation, we calculated the square root of the sum of squares, and then calculated the ratio of the binocular summation (prediction/measured). The average ratio for all durations was 1.15 ± 0.09 (60,1.16 ± 0.1; 120,1.13 ± 0.08; 240,1.09 ± 0.06; 320,1.11 ± 0.1; 480,1.25 ± 0.11; mean ± SE) denoted by a dashed blue line (Fig. 2). These ratios indicate that actual binocular viewing followed the expected quadratic summation of the two monocular inputs. Hence, this implies that there was neural binocular summation, which was confirmed not to be a statistically significant different between the prediction and the measured (p = 0.07), by 2-way ANOVA with eyes (prediction/measured) and presentation time [ms] (60, 120, 240, 320, and 480). For more temporal statistical information, see Table 4.

### Contrast sensitivity between eyes

The visual acuity difference between the eyes was minor in normally sighted subjects 0.01 logMar (Table 3). Thus, to test the contrast sensitivity result s as poor-better eyes, the contrast sensitivity results were separated according to the dominant and non-dominant eye (Table 4).

Trend lines for binocular, dominant, and non-dominant eyes for the five durations were calculated and are denoted by blue, magenta, and black lines, respectively. Again, contrast sensitivity increases as a function of presentation time. The critical duration of binocular, dominant, and non-dominant eyes were 196, 250, and 294 ms, respectively.

The dominant/non-dominant eye ratio was 1.08 ± 0.14. No statistically significant differences were found p = 0.36, as confirmed by 2-way ANOVA with eyes (Dominant/Non-dominant) and presentation time [ms] (60, 120, 240, 320, and 480), see Table 4. Binocular summation was calculated for each time condition for both the dominant and non-dominant eyes. The average binocular summation is 40 ± 13%. For more temporal statistical information, see Table 4.

### Differences between controls and nystagmus subjects

When comparing the results of the strong and poor eyes of IN and normally sighted subjects, we observed decreased contrast sensitivity in IN by an average factor of 1.27 ± 1.55 for the better eye and a factor of 1.5 ± 0.18 for the poor eye. Critical durations of normally sighted subjects were shorter than those of IN. The fastest critical duration was for the binocular condition, followed by the dominant/better eye, and the least for the non-dominant/poor eye.

## Discussion

### Monocular viewing in infantile nystagmus

In this study we found differences in contrast sensitivity between monocular R and L eye viewing, showing a ratio of 1.9 ± 0.12 of better/poor eyes, which is larger than the ratio found in normally sighted subjects and remarkably and significantly different from the same calculation in the control (p = 5.2062e-04). This finding was also observed in visual acuity differences of 0.1 ± 0.02 (mean ± SE) logMar (one line) between the eyes (for both far and near distances) and was compatible with the subjects’ dominant eye.

Amblyopia is defined as a difference in visual acuity between the two eyes of two lines or more^52^. Here we found a decrease in visual acuity in both monocular and binocular viewing (measured); since the difference between the eyes was no more than one ETDRS line, our IN subjects are not considered amblyopic. In addition, there are some differences in visual acuity between eyes in normally sighted subjects (0.04–0.08 logMar); however, we did not find a difference in contrast sensitivity between eyes, which we found in IN subjects. Hence, we can conclude that what influences the contrast sensitivity differences between eyes was not only the visual acuity. Moreover, when we examined the results without NYS-1, which might have been considered as an outlier because of his good visual acuity, the superior effect of the better eye was still maintained. Many studies have shown that contrast sensitivity is a more sensitive indicator of fine impairment changes than visual acuity is^14,15^. It is known that amblyopia affects more visual functions than just visual acuity^27–31^. Thus, we suggest that the conservative definition of amblyopia based on  visual acuity alone might be narrow and may miss cases of amblyopia where the differences (between the eyes) of visual acuity are small but the contrast sensitivity is large. Such cases can be viewed in our IN subjects, which are not defined as amblyopia but present features similar to amblyopia”.

Previous studies that investigated visual performance such as visual acuity and crowding in IN had distinguished between poor and better eyes. They found better acuity and less crowding effect in the better eye of IN^50,51^. Other studies that investigated contrast sensitivity in IN were performed under either binocular viewing^47,53^ or under only the dominant eye^54^; hence, a direct comparison of our results of contrast sensitivity to these reports is not possible.

Studies have suggested that the presence of eye dominance in normally sighted subjects implies some sort of binocular inhibitory interaction, in which the image originating from the non-dominant eye is suppressed during binocular vision^55,56^. In the normally sighted population, the existence of a dominant eye is controversial. A study on normally sighted subjects found some temporal preference of the dominant eye over the non-dominant eye, showing faster information processing (however, not statistically significant differences)^57^. In addition, during binocular rivalry tasks, the amount of time the target is perceived via the dominant eye is greater than the duration of the non-dominant eye^58^. Studies suggest that the duration of a signal in the dominant eye is prolonged, thus increasing the sensitivity reduction in the non-dominant eye^55,56^. We suggest that for some abnormal developmental reason the signals received from the dominant eye is leading to weaker processing of contrast sensitivity and visual acuity of the poor eye compared with the better eye in IN. Moreover, we hypothesize that the strong effect we found can occur by several developmental impairments, when the underlying mechanisms originates from strabismus^50,59^, astigmatism^41,50,54,60^, anisometropia^60^, refractive errors, and features of eye movements such as nystagmus amplitude, frequency^50,61^, and foveation time^38,41,53,62,63^.

### Strabismus

An additional parameter of interest that affects amblyopia is strabismus. Strabismus in IN is very prevalent^41,64^. In our study we found that seven out of ten IN subjects had strabismus. Four of them had constant strabismus; the rest had time intervals in which there was no misalignment (intermittent tropia) or the misalignment alternated between the eyes (alternating tropia). To investigate the effect of strabismus on contrast sensitivity, we excluded from the average the results of IN subjects with a constant strabismus. The results show a slight reduction in the ratio of better/poor eyes (from 1.46 to 1.4); however, the significant difference between better-poor eyes was maintained (p = 0.0026, n = 6). When examining the results of the three subjects with no strabismus, the best eye-poor eye ratio was 1.46 and the difference was significant (p = 0.03, two-way ANOVA, eye (better, poor) and time presentation (60, 120, 240, 320, and 480)). Hence, strabismus per se cannot account for our results.

### Refractive errors and anisometropia

The existence of large refractive errors in IN was previously reported in the literature^41^. In our study all IN subjects needed correction with at least one of the refractive errors such as hyperopia or myopia; however, astigmatism was most prevalent in both eyes. Cho *et al*. (2009) carried out a study focusing on the dominant eye in normal populations and reported that refractive errors can affect the development of a dominant eye. The astigmatic component of refractive errors has a greater impact than spherical myopia. In addition, accommodative control is superior in the dominant eye^60^. Low-vision subjects frequently report that spectacles do not improve their vision and hence, refuse to wear them, similarly to reports in subjects with amblyopia. Despite subjective behavior regarding spectacle wearing, studies showed that proper correction of vision is crucial, especially when nystagmus is concerned^41^ in which a reduction of nystagmus eye movement was reported^65^. In our study three out of ten IN subjects had never been fitted with a refractive correction; hence, impairment of cortical developmental resulting from uncorrected refractive error is plausible. This, however, cannot explain the differences between the eyes.

### Eye movements

Eye movements are considered to be the underlying cause of vision deterioration in albino and especially in idiopathic nystagmus^41,61^. Hence, in this experiment the size of the Gabor target was designed to be larger than the expected saccade amplitude in our nystagmus subjects, with the aim to avoid missing targets due to eye oscillations.

In addition, in our study eye movements were recorded using Tobii technology ab (Sweden) with a sampling rate of 90 Hz. Subjects passively viewed a white circle representing the target at a visual angle of 0.43° for ten seconds, with six repetitions. Our results [Vision Science Society, 2019, 35.435] showed that no correlation was found between the subjects’ visual acuity and other functional parameters of eyes that we examined, or with the frequency or amplitude of saccades, or with the scatter position.

Moreover, we found that oscillation of eye movements did not predict the degree of impairment or differences between eyes, consistent with previous studies that found that a decrease in oscillation parameters (in convergence and null point) did not improve vision^41,47,48^. Additional studies found an amblyopic component in idiopathic IN, which is mainly responsible for the visual impairment, and which does not relate to eye movements^47,65^.

Thus, we suggest that eye movements are not the main cause of the abnormal features we found in IN; rather, they are indicative of impaired developmental processing in the critical period for both the visual system and for early oculomotor plasticity^66^.

More elaborate eye movement results are planned to be published in a separate follow-up paper.

### Binocular summation

Early studies of binocular summation found an empirical improvement of a factor of about 1.4, leading to models assuming a quadratic summation of the two monocular inputs (√2)^16,67–73^. A recent review by Baker *et al*. (2018) showed that different amounts of binocular summation exist in a range from √2 to 2 values^17^ because the amount of binocular summation is affected by the spatiotemporal parameters of the stimulus. Ding and Sperling (2005), in their study on binocular summation, suggested a gain control theory based on studies by Cogan (1987) and Wilson (2003). They suggested that each eye can exert gain control on the other eye’s gain control in a closed loop manner; hence, unidentical contrast from one eye exerts gain control on the other eye, depending on the robustness of its own input. This resulted in unequal contributions to binocular summation and reduced it^71^. This effect is noted empirically with amblyopic eyes^16,23,56,72,74^. The conventional paradigm regarding binocular summation in amblyopia is that there is a lack of neuronal summation^18,21,56,75,76^. However, Baker *et al*. (2007) found binocular summation in a neural convergence mechanism in strabismus amblyopic subjects based on the calculation of $summation=\sqrt{R\,ey{e}^{2}+L\,ey{e}^{2}}$^23,77^. However, the authors did not claim that this phenomenon generally occurs for all cases of amblyopia^23^.

In our study the average ratio of the binocular summation (expected/measured) was 1.27 (p = 0.04). The control group showed an average ratio of 1.15 (p = 0.07), meaning that the difference between expected to the actual measure is less different. Hence, the difference between the two groups indicates that there was a residual effect of neuronal summation in IN but less than expected. While inspecting the individual data, four out of the ten subjects’ results indicated that their neural mechanism of summation remained intact. Thus, apparently this topic needs further investigation.

We noted that regardless of whether summation occurs or not, the measured binocular contrast sensitivity in nystagmus subjects resembles more the better eye (1.2 ratio) than the poor eye (2.17 ratio). This finding is reminiscent of previous studies in amblyopic sujects^21,23,56^. However, the measured binocular summation in the control group was as expected, better from the strong and poor eyes by about 40%.

### Fusion

**Figure 3:**
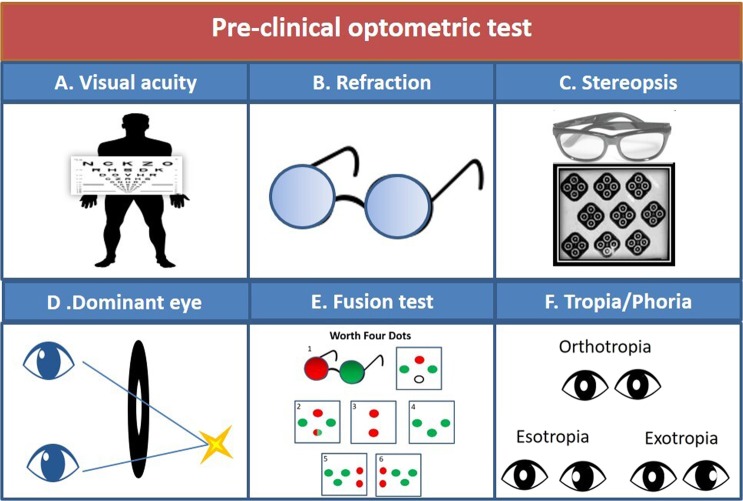
Pre-clinical optometric tests. (**A**) Monocular and binocular visual acuity were measured for three distances. (**B**) Refractive corrections. (**C**) Stereoscopic vision exam. (**D**) The dominant eye was evaluated by the forcing monocular vision technique. (**E**) Fusion tests were performed using the Worth Four Dots test. **E1**. The subject wears red green spectacles and watches a target consisting of 4 colored dots. The subject can report: **E2**. Fusion. **E3**. Suppressing the left eye. **E4**. Suppressing the right eye. **E5**. Uncrossed Diplopia, Eso deviation. **E6**. Crossed Diplopia, Exo deviation. (**F**) Binocular motility was determined with the ‘cover test’ method. Occlusion of the good eye revealed the type and direction of Tropia.

Fusion is a clinical condition that can affect binocular contrast sensitivity and specifically binocular summation. Fusion occur also when the two images received from the eyes are slightly spatially displaced and still interpreted subjectively as one image. We determined fusion using the Worth four-dot test (with a different color lens presented to each eye (Fig. 3)). When we compared the contrast sensitivity of the binocular results of the four nystagmus subjects, which showed fusion, to the other six nystagmus subjects without fusion, their binocular condition was not significantly different. This is true for the measured (p = 0.96) and the predicted (p = 0.83) [see the Supplementary for statistical information (Table S1) and the contrast sensitivity **(**Fig. S2)].

### Temporal contrast sensitivity and critical duration

Bloch’s law suggests that contrast sensitivity increases when the duration of the stimuli is prolonged^78^. However, this law is only valid for short durations down to several hundreds of milliseconds^79,80^. For longer durations, temporal summation is limited. Additional support for the superiority of binocular viewing is provided by our findings of a shorter critical duration under binocular conditions, which indicates faster visual processing. The IN and normally sighted subjects exhibit a shorter critical duration under binocular conditions, compared with each eye separately (even shorter than the better eye), which indicates a shorter integration time. In addition, the individual results of the poor eye showed relationships between the critical duration and contrast sensitivity of the poor eye.

Examining the temporal aspect of visual processing in subjects that experience continuous instability of the retinal image is very important in deciphering and understanding visual function in nystagmus. Previous studies investigating IN mainly focused on static spatial functions of the visual system such as visual acuity^42^, crowding^43^, and CSF (contrast sensitivity function)^54,81^. Here we focused on the temporal aspect of the visual system and binocular summation. To the best of our knowledge, this is the first comprehensive study that investigates temporal contrast sensitivity in IN subjects.

In a previous study in normal sighted poplulation, Loshin *et al*. (1982) used a grating target at spatial frequencies of 0.5–8 cpd with presentation times between 20 and 4000 ms and found that critical duration is between 90 and 180 ms^19,82,83^. They suggested that critical duration is prolonged due to poor fixation as in amblyopic subjects, resulting in a longer critical duration^19^.

As previously mentioned, when investigating how time influences contrast sensitivity, we found an increase in contrast sensitivity as a function of the longer presentation time of the target for both normally sighted and IN subjects, consistent with previous studies^24,84^. However, whereas IN continued to improve in contrast sensitivity up to the longest presentation time of 480 ms in the poor eye, normally sighted subjects reached saturation at earlier times and under all conditions. We found that critical duration has a hierarchy, with the fastest duration being for binocular, then the better eye, and the slowest duration for the poor eye for both IN and normally sighted subjects. These results suggest that the poor eye contributes to the superiority of the binocular condition, even with significant differences between the eyes in cases such as IN.

The critical duration of IN under monocular and binocular conditions was longer for mal-sighted subjects by a factor of 1.88, 1.55, and 1.56 ms for binocular, the better eye, and the poor eye. The critical duration of the poor eye in IN was 470 ms, in agreement with the finding of a critical duration of 475 ms in the amblyopic eye for spatial frequency of 8 cpd^19^. The dashed blue lines in Fig. 2 suggest that even if subjects with IN were able to optimally integrate the inputs from the two eyes, their critical duration would still be longer than normal. By contrast, the critical duration of normally sighted subjects was much shorter, 196 ms. This finding is consistent with previous studies on normally sighted subjects, which found a saturation of contrast threshold detection at 160–200 ms for the binocular condition^82,83^. However, data on monocular critical duration are rare.

### Spatial frequency

A previous study suggests that probably the most important features influencing the CSF are the bandwidth of spatial frequency and the temporal waveform^85^. Accordingly, in our study, the spatial frequencies of the Gabor patches were adjusted such that they were slightly above the cutoff threshold (better) for each subject. The cutoff frequencies that we found are comparable with those of previous studies, revealing the impaired spatial frequency performance in nystagmus subjects, compared with normally sighted subjects^47,54,81^. In our study, the IN subjects exhibited deterioration in monocular and binocular performance, and low spatial frequency when they adjusted to the IN subjects (2.75 ± 2.2, mean ± SD). This was predicted from the literature. This finding is thought to be attributed to eye oscillations, leading to blurring of the image formed on the retina^41,47^.

The reduction in cutoff frequency in IN, compared with normally sighted subjects, is accompanied by a reduction in contrast sensitivity. It resembles strabismus amblyopia more than anisometropic amblyopia, which affects both low and high frequencies^19,86,87^.

It is difficult to recruit congenital nystagmus subjects which is unique case (1/5000) and that occasionally associate with albino that may suffer from pathology that affect sight, thus we had to exclude some of them from the study. Moreover, there was complaints issue: due to the demanding testing in our study of the subjects, which was distributed over a few days, and the few hours for each visit; some subjects couldn’t complete the required number of testing sessions; thus, they were not included in the final data. Therefore, only ten nystagmus subjects participated in the experiment. The variability between subjects include different eye movement features (amplitude and frequency) and visual performance such as visual acuity, spatial frequency cutoff, sensitivity and difference in the integration time needed for visual information (critical duration) and binocular summation. However intriguingly, despite the variability between subjects, they still showed the effect of different performance between eyes (better vs. poor eye).

### Summary

In this research we thoroughly investigated monocular and binocular contrast sensitivity in patients with IN and the integration time needed for extracting visual information. We found a strong asymmetry between the eyes, creating an effect of poor-better eyes in IN subjects. Normally sighted subjects exhibited superiority (summation) in contrast sensitivity under the binocular condition, whereas only some IN subjects exhibited some degree of superiority in binocular viewing. In IN subjects, an additional difference was observed: a longer integration time is needed to reach saturation in processing visual information.

## Methods

The experimental protocol was approved by the internal board of the ethics committee (IRB) of Bar-Ilan University, according to the guidelines and regulations for human subject research. All experimental protocols were performed in accordance with the guidelines provided by the committee approving the experiments. The participants were recruited using electronic advertisements, public advertisements, and direct recruitment, and signed a consent form and filled out a medical questionnaire. All participants signed an informed consent and received monetary compensation for their time and travel for participating in the study.

### Participants

A total of 20 adult subjects participated in the study. The study group included subjects with Infantile nystagmus (IN, n = 10; idiopathic IN, n = 2; oculocutaneous albinism accompanied by nystagmus, n = 8) and a control group with normal vision (n = 10). The age of the nystagmus subjects was between 19 and 43 years (29.9 ± 6.9, mean ± SD). The age of the control group was between 23 and 37 years (mean of 28 ± 4). The nystagmus and the normally sighted groups were aged matched (t-test, p = 0.464). Study inclusion criteria for the nystagmus group were an age range from 18 to 45 years old, with no prior eye surgeries; in addition, the direction of the nystagmus was horizontal. Nystagmus etiology is congenital, during the first few months after birth, and is diagnosed in early childhood by an ophthalmologist. Thus, one subject with acquired nystagmus due to cornea dystrophy and with no manifestation of other ocular pathologies was excluded prior to testing. Nystagmus subjects that fulfilled the above criteria and took part in the study were required to repeat the experimental tasks at least twice, on two separate days. Inclusion criteria for normally sighted control group was visual acuity that is not worse than 0.0 logMar (6/6) monocular and binocular and the difference between eyes is not more than 0.1 log unit (one ETDRS line).

### Apparatus

We utilized a customized platform for psychophysical and eye-tracking experiments PSY, which was developed by Dr. Yoram S. Bonneh^88^. The stimuli consisted of Gabor patches that were displayed using an Eizo, FG-2421, 24”, HD monitor running at 120 Hz, which overcomes display time uniformity issues faced by other LCD monitors and is therefore suited to psychophysics exams^89^. The effective size of the monitor screen was 52 by 30 cm, with a resolution of 1920×1080 pixels. The screen was calibrated and Gamma correction was set to 2.91. The experiments were performed with a background luminance of 40 cd/m^2^.

### Procedure for optometric tests

The procedure in the study includes comprehensive clinical well-established optometric methods^90–93^ (Fig. 3) by a qualified optometrist (A.M). The tests included a refractive test, static visual acuity for 40 cm, 60 cm, and 3 m (Fig. 3A), refraction (Fig. 3B), stereoscopic acuity using the ‘Stereo-fly test’ (Fig. 3C), a fusion test with a ‘Worth 4 Dots’ test (measures the subjective binocular perception and evaluates the properties of binocular vision; the evaluation can be normal vision, or in cases of strabismus, suppression, and/or diplopia (Fig. 3D)). In addition, the presence, direction, and magnitude of the phoria or tropia (latent or manifest, respectively, misalignment of the eyes (Fig. 3D)) was determined through horizontal and vertical prism-bars, using the ‘Cover test’ method performed by two qualified optometrists (A.M and I.Z). The dominant eye was determined by forcing monocular viewing by looking through a hole in a card, or through a hole made with subjects’ hands (Fig. 3D). All subjects were evaluated.

### Contrast sensitivity experiment

Both the study and control groups participated in similar detection contrast sensitivity experiments, with the stimulus parameters adjusted for each subject in the IN group suited to their low vision. Monocular and binocular vision were obtained using right and left opaque lenses with a blocking power of 99.5 percent. Target stimuli were localized gray-level vertical Gabor patch stimuli. The stimulus contrast is defined by the Michelson formulation^94^: $\frac{{\boldsymbol{Imax}}-{\boldsymbol{Imin}}}{{\boldsymbol{Imax}}+{\boldsymbol{Imin}}}$. It was shown that in both cases of nystagmus associated with albino^54^ and for idiopathic nystagmus^95^, the resolution increases and the spatial frequency cutoffs were higher for horizontal than for vertical gratings, when the orientation of the grating is parallel to the meridian of the nystagmus. The study of Bedell and Loshin^54,95^ tested in nystagmus if the spatial frequency cutoff at different orientations (horizontal and vertical meridians) were affected by the horizontal eye movements, hence affecting visual acuity. They found that visual acuity correlates significantly with the cutoff in both orientations^54,95^. In our study, we chose to use vertical Gabors because studies in our lab (e.g. Yehezkel Ph.D. Thesis, 2012) found that sensitivity to vertical Gabor is higher in monocular than in binocular viewing, but it might be reversed in the horizontal condition mainly in clinical cases. Since in this study we compared the sensitivities of monocular and binocular conditions, we believe that this approach provided more reliable data.

**Figure 4:**
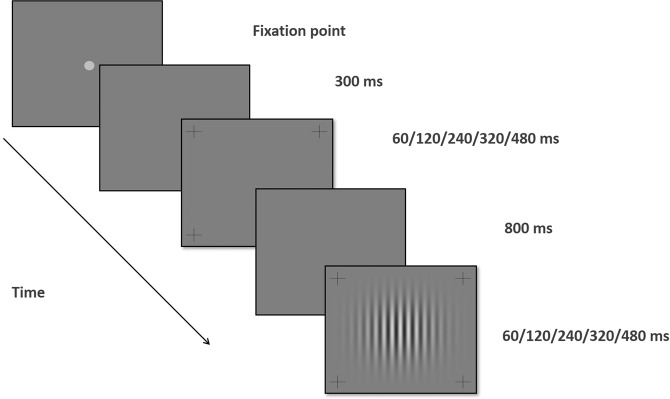
Two alternative forced choice paradigms of the experiment and time windows. At 300 ms after the fixation point disappears, a window in which a Gabor patch stimulus (GPs) may appear is presented for one of the five durations determined in the block. After 800 ms, the second other alternative, where GPs may appear, start. The duration of the window is constant and is determined by the block chosen before.

The Gabor patches’ contrast level was modulated from a background luminance of 40 cd/m^2^. The experiment was divided into five temporal blocks; in each block, the duration was constant: 60, 120, 240, 320, or 480 ms. Contrast sensitivity threshold under each condition was evaluated using the two temporal alternative forced choice (2TAFC) paradigm. A visible fixation circle in a visual angle of 2 degrees was presented in the center of the screen until the participants pressed the button to start the stimulus presentation. The order of presentations was as follows: Once the subject pressed the button, a 300 ms blank period with a temporal jitter of 500 ms, on average, followed the fixation point. Both the target and non-target options had the same presentation duration (60, 120, 240, 320, and 480) with time intervals of 800 ms between them. The target Gabor patches were presented in only one of the two intervals (the order was randomized). Participants were asked to report which interval contained the target by pressing a mouse button (left for the first interval and right for the second). Across trials, the target presentation was equally distributed between the two intervals. Participants were instructed to maintain their fixation at the center of the monitor and to avoid eye movements during the trials (Fig. 4). Contrast thresholds were then determined utilizing a 3:1 staircase method, which was shown to converge to 79% correct^96^. In this method, the target contrast is increased by 0.1 log unit (26%), after an erroneous response, and is decreased by the same amount after three consecutive correct responses^26,96^. Audio feedback is provided to the subject after an incorrect response.

Stimulus size was set to σ = 400 pixels, which at a viewing distance of 60 cm, suspends a visual angle of 10 degrees. Low-vision subjects adopt techniques to improve their vision; they tend to look at the target from shorter distances to increase the angular magnification. In line with this approach, we used a chin rest in order to restrict the continuous changes in body and head posture. Spatial frequencies of the Gabor patches were adjusted specifically for each nystagmus subject; then they were fixed for all sessions and task time condition. Spatial frequency was determined in the first or the second meeting. The experiment started with a training session in which high contrast with low spatial frequency Gabor patches were presented in order to ensure that the subject understood the task. Once this was achieved, for each subject, we determined the spatial frequency threshold at a contrast of 80 for the shortest duration (60 ms). Subjects that did not detect the Gabor patches of a spatial frequency of 1 cpd moved to a lower spatial frequency of 0.5 cpd. Normally sighted spatial frequencies of the Gabor patches were set to 9 cpd for all subjects, which was the most reliable spatial frequency we could obtain. This is due to the sitting distance limitation and the requirement to induce the same Gaussian decline as in IN subjects. This spatial frequency, which was presented to normally sighted subjects, was found in a former study to be close to the limit for testing the visual system using Gabor patches^82^.

The block order of the IN experiment began in each session with one of the longer presentation times (480 or 320 ms) to decrease the visual stress of the subject when performing the fast durations. The remaining blocks were presented randomly. Ten to 15-minute breaks were given between two to three blocks. Subjects repeated all five blocks for the monocular and binocular condition, on the same day. The subjects repeated the task four times on four separate days. Control subjects repeated all five blocks consecutively in one session and repeated the measurements on two separate days.

### Eye movement experiments

In order to evaluate the ability to maintain fixation, damaged by the involuntary oscillation movements caused by nystagmus, under the continuous presentation of static targets, the subjects were required to passively watch a white circle that served as a target projecting at a visual angle of 0.47°. The background was set to black. The target was localized to the center of the screen for ten seconds; each session consisted of six repetitions interleaved with rest periods chosen by the subjects to reduce subject fatigue. Each subject repeated the target twice, at each meeting. We used the Tobii 4 C device with a sampling rate of 90 Hz (the EyeLink 1000 infrared system was inadequate for reading the albino subject’s eye movements) and for this set of experiments the stimuli were displayed on a BenQ, XL 2411, 24″, 3D ready monitor, running at 60 Hz with an effective monitor size of 53.5 by 30 cm and at a pixel resolution of 1920 × 1080.

### Data analyses

First, the repetitions for each subject for each presentation condition were averaged. Next, the average contrast sensitivity data were fitted to an exponential curve using a custom-written MATLAB code (MATLAB 2014b The Mathworks, Waltham, Massachusetts), specifically using the ‘feminsearch’ function. The curve parameters were estimated using the least-mean square error as a measure. The curve to which the data were fit is ${\rm{Ca}}={\rm{C}}1\ast ({\rm{C}}2-{{\rm{e}}}^{-\frac{{\rm{t}}}{{\rm{\tau }}}}\,)$, where t is the presentation duration in milliseconds, C1*C2 is the asymptote, and τ is the time constant.

All results were normally distributed and statistical analyses were performed using the two-way ANOVA MATLAB tool.

Student’s t-test analyses were performed using Microsoft Office Excel 2007.

## Supplementary information

Supplementary information
