# Supplementary material for: Monocular and Binocular Temporal Visual Perception of Infantile Nystagmus
Source: Sci Rep. 2020 Mar 18;10:4946. doi: 10.1038/s41598-020-61914-2 (PMC7080729; doi:10.1038/s41598-020-61914-2)
Supplement: Supplementary file 1 — Supplementary information [file 41598_2020_61914_MOESM1_ESM.pdf]

Monocular and Binocular Temporal Visual Perception of Infantile Nystagmus

Avital Moshkovitz, Maria Lev, and Uri Polat.

School of Optometry and Vision Sciences, Faculty of Life Sciences, Bar-Ilan University.

Supplementary

All individual contrast sensitivity results presented in **fig s.1**. Critical duration presented as triangular on the corresponding curve in the graph.

Results of 4 nystagmus subjects (NYS-1, NYS-3, NYS-5 and NYS-7) presented binocular summation equal or better to the binocular viewing performance. Even though the neural summation were different between subjects, all subjects exhibit priority of the better eye.

For investigating binocular fusion effects on binocular summation, we compare results of fusion and non-fusion results base on Worth Four Dot test presented in **fig s.2**. The differences between fusion binocular contrast sensitivity results and non-fusion binocular contrast sensitivity results, and in addition the comparison between the expected binocular summation of both group were not statistically significant different (See **table s.1**) statistical analyses, performed using the N-way analysis of variance with ANOVA MATLAB tool.

Eye / Time	Binocular fusion	Binocular non-fusion	ANOVA	Calculated Binocular fusion	Calculated Binocular non- fusion	ANOVA
**480**	1±0	1±0	0.96	1.35±0.4	1.2±0.12	0.83
**320**	0.93±0.07	0.85±0.08		1.24±0.28	1.0±0.14	
**240**	0.95±0.16	0.77±0.04		0.72±0.12	0.95±0.14	
**120**	0.46±0.09	0.52±0.07		0.66±0.18	0.72±0.06	
**60**	0.33±0.09	0.38±0.06		0.45±0.2	0.47±0.06	

Table s.1 *Statistical and average contrast sensitivity of subjects that report on fusion in Worth four Dots exam (n=4) and IN subjects that did not report on fusion in Worth four Dots exam (n=6). Binocular refer to binocular condition when both eyes were open. Calculated binocular refers to the square root calculation of binocular summation*

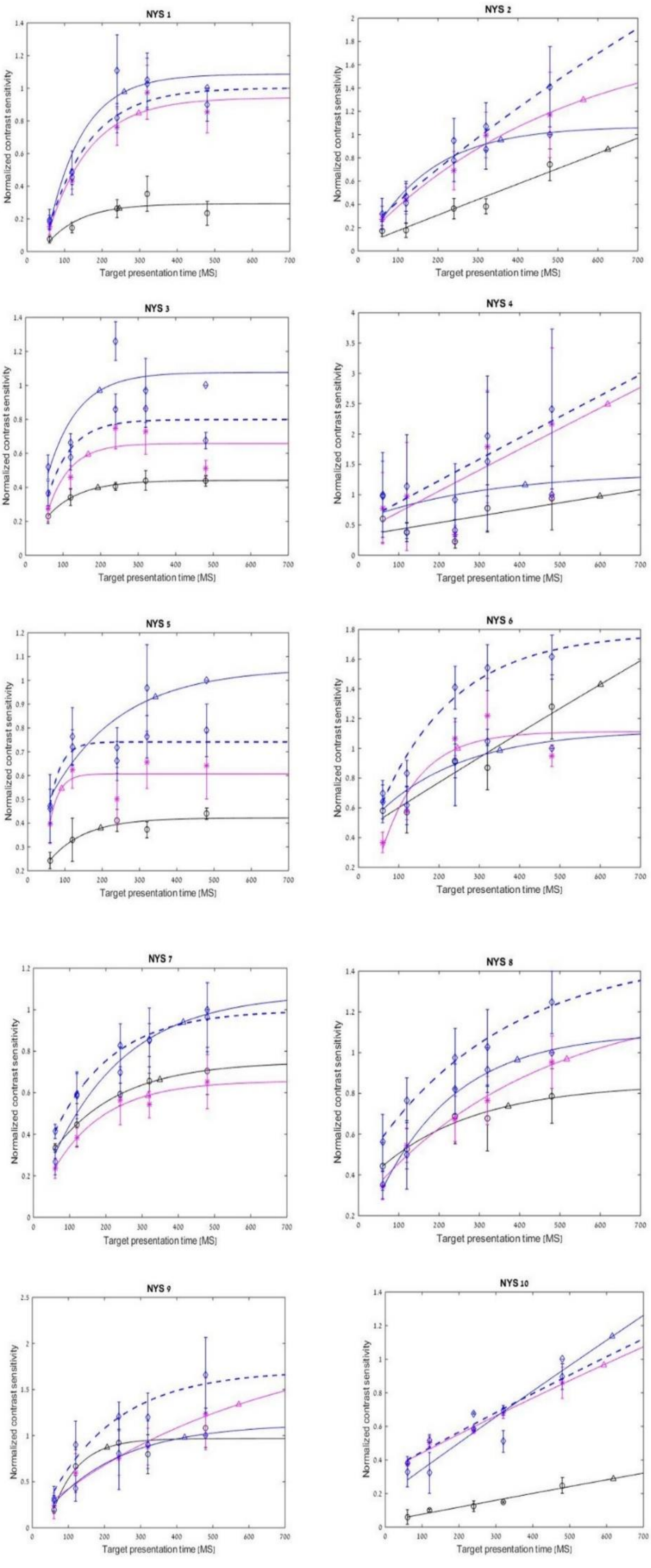
NYS 1
Normalized contrast sensitivity
Target presentation time [MS]
NYS 2
Normalized contrast sensitivity
Target presentation time [MS]
NYS 3
Normalized contrast sensitivity
Target presentation time [MS]
NYS 4
Normalized contrast sensitivity
Target presentation time [MS]
NYS 5
Normalized contrast sensitivity
Target presentation time [MS]
NYS 6
Normalized contrast sensitivity
Target presentation time [MS]
NYS 7
Normalized contrast sensitivity
Target presentation time [MS]
NYS 8
Normalized contrast sensitivity
Target presentation time [MS]
NYS 9
Normalized contrast sensitivity
Target presentation time [MS]
NYS 10
Normalized contrast sensitivity
Target presentation time [MS]

Fig s.1 *Normalized contrast sensitivity for Infantile nystagmus (IN) for varying presentation times. The fitting curve is described in the Methods section. Each line is a fit to the monocular or binocular conditions of measured contrast sensitivity. Binocular, better, and poor eye are denoted in blue, magenta, and black solid lines, respectively. Dashed blue line presents the square root calculation of binocular summation (√2). Error bars refer to the standard error of the mean. Critical duration is denoted by triangular symbols in corresponding colors.*

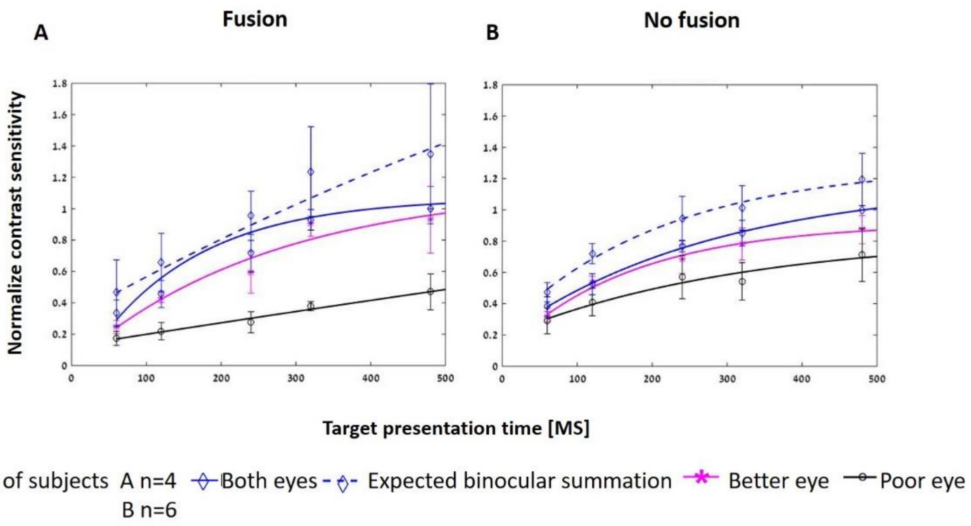

Fig s.2 *Normalized contrast sensitivity for Infantile nystagmus (IN) for varying presentation times. A. IN subjects that report on fusion in Worth four Dots exam (n=4). B. IN subjects that did not report on fusion in Worth four Dots exam (n=6). Binocular, R, L, better, and poor eye are denoted in blue, magenta, and black solid lines, respectively. Dashed blue line presents the square root calculation of binocular summation (√2). Error bars refer to the standard error of the mean. Critical duration is denoted by triangular symbols in corresponding colors.*
